# Primary pyogenic spondylitis following kyphoplasty: a case report

**DOI:** 10.1186/1752-1947-5-101

**Published:** 2011-03-13

**Authors:** Markus D Schofer, Stefan Lakemeier, Christian D Peterlein, Thomas J Heyse, Markus Quante

**Affiliations:** 1 Department of Orthopaedics, University Hospital Marburg, Baldingerstrasse, 35033 Marburg, Germany

## Abstract

**Introduction:**

Only ten cases of primary pyogenic spondylitis following vertebroplasty have been reported in the literature. To the best of our knowledge, we present the first reported case of primary pyogenic spondylitis and spondylodiscitis caused by kyphoplasty.

**Case presentation:**

A 72-year old Caucasian man with an osteoporotic compression fracture of the first lumbar vertebra after kyphoplasty developed sensory incomplete paraplegia below the first lumbar vertebra. This was caused by myelon compression following pyogenic spondylitis with a psoas abscess. Computed tomography guided aspiration of the abscess cavity yielded group C *Streptococcus*. The psoas abscess was percutaneously drained and laminectomy and posterior instrumentation with an internal fixator from the eleventh thoracic vertebra to the fourth lumbar vertebra was performed. In a second operation, corpectomy of the first lumbar vertebra with cement removal and fusion from the twelfth thoracic vertebra to the second lumbar vertebra with a titanium cage was performed. Six weeks postoperatively, the patient was pain free with no neurologic deficits or signs of infection.

**Conclusion:**

Pyogenic spondylitis is an extremely rare complication after kyphoplasty. When these patients develop recurrent back pain postoperatively, the diagnosis of pyogenic spondylitis must be considered.

## Introduction

Vertebroplasty and kyphoplasty are discussed critically in the literature [1-6]. The overall risks of these procedures are low and more severe complications such as spinal cord compression or pulmonary embolism are very rare (0.01%-0.03%) after kyphoplasty [2]. Older patients undergoing kyphoplasty may have risk factors for immunocompromise, such as diabetes or renal insufficiency. Until now, there have been no reported cases of primary pyogenic spondylitis or spondylodiscitis after kyphoplasty.

## Case presentation

**Figure 1:**
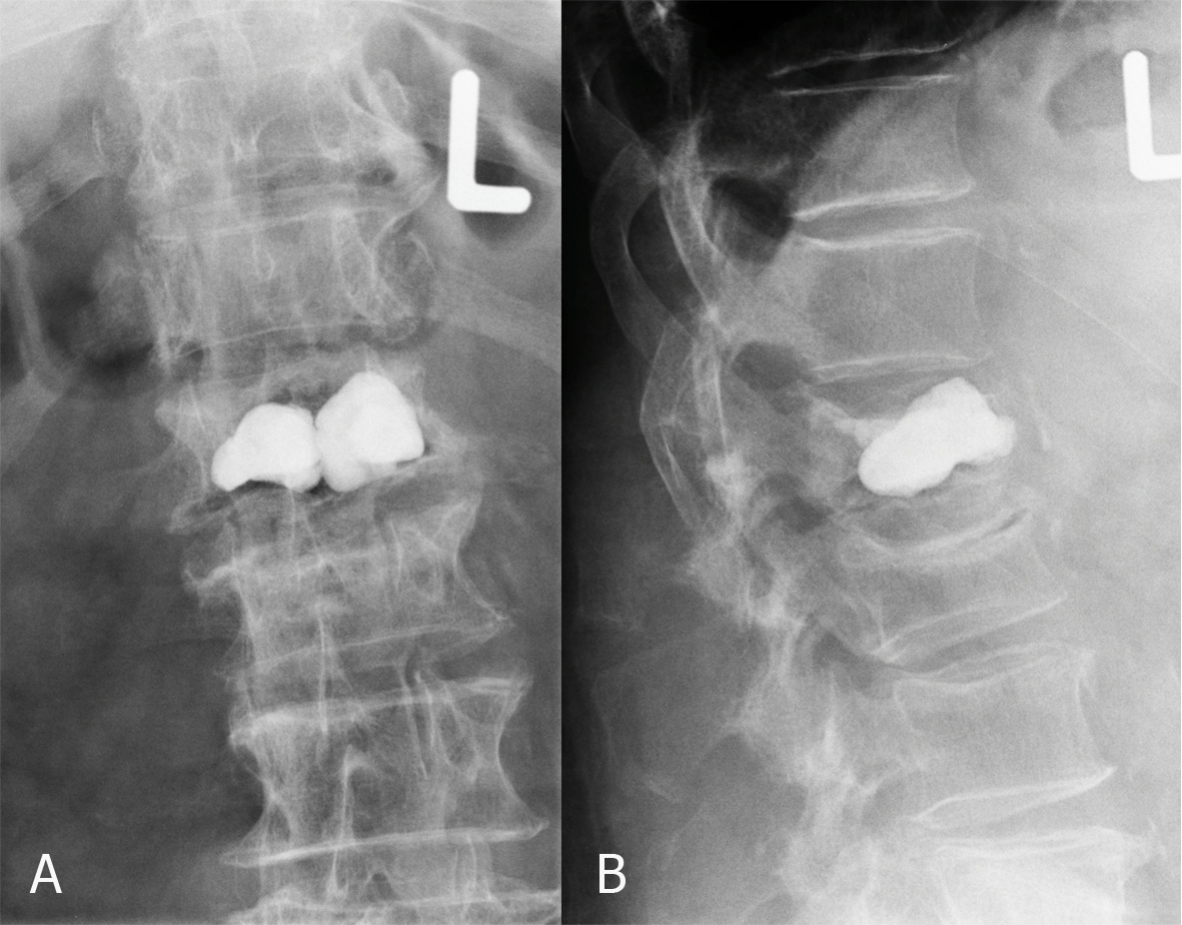
**Plain (A) and lateral (B) thoracolumbar radiographs (T11 - L3) taken after initial kyphoplasty for treatment of an L1 compression fracture**. The cement is correctly positioned in the vertebral body.

A 72-year-old Caucasian man, with a past medical history of mild Parkinson's disease, hypertension, coronary artery disease and cardiac insufficiency, complained of four weeks of back pain. Physical examination and imaging with computed tomography (CT) and magnetic resonance imaging (MRI) revealed a recent osteoporotic compression fracture of L1 and an older, consolidated fracture of the L2 endplate. The patient underwent the initial operation at an outside institution; bilateral transpedicular L1 kyphoplasty was performed, using the Kyphon^® ^(Sunnyvale, CA, USA) kyphoplasty system with polymethylmethacrylate cement. A single dose of antibiotic prophylaxis (cefazolin sodium USP, 2 g) was administered preoperatively. Intraoperatively, a bone cylinder biopsy was taken; histological examination showed no evidence of malignancy or infection. Plain radiographs demonstrated satisfactory placement of the cement in the vertebral body (Figure 1). He was discharged on the postoperative day six pain free and neurologically intact.

**Figure 2:**
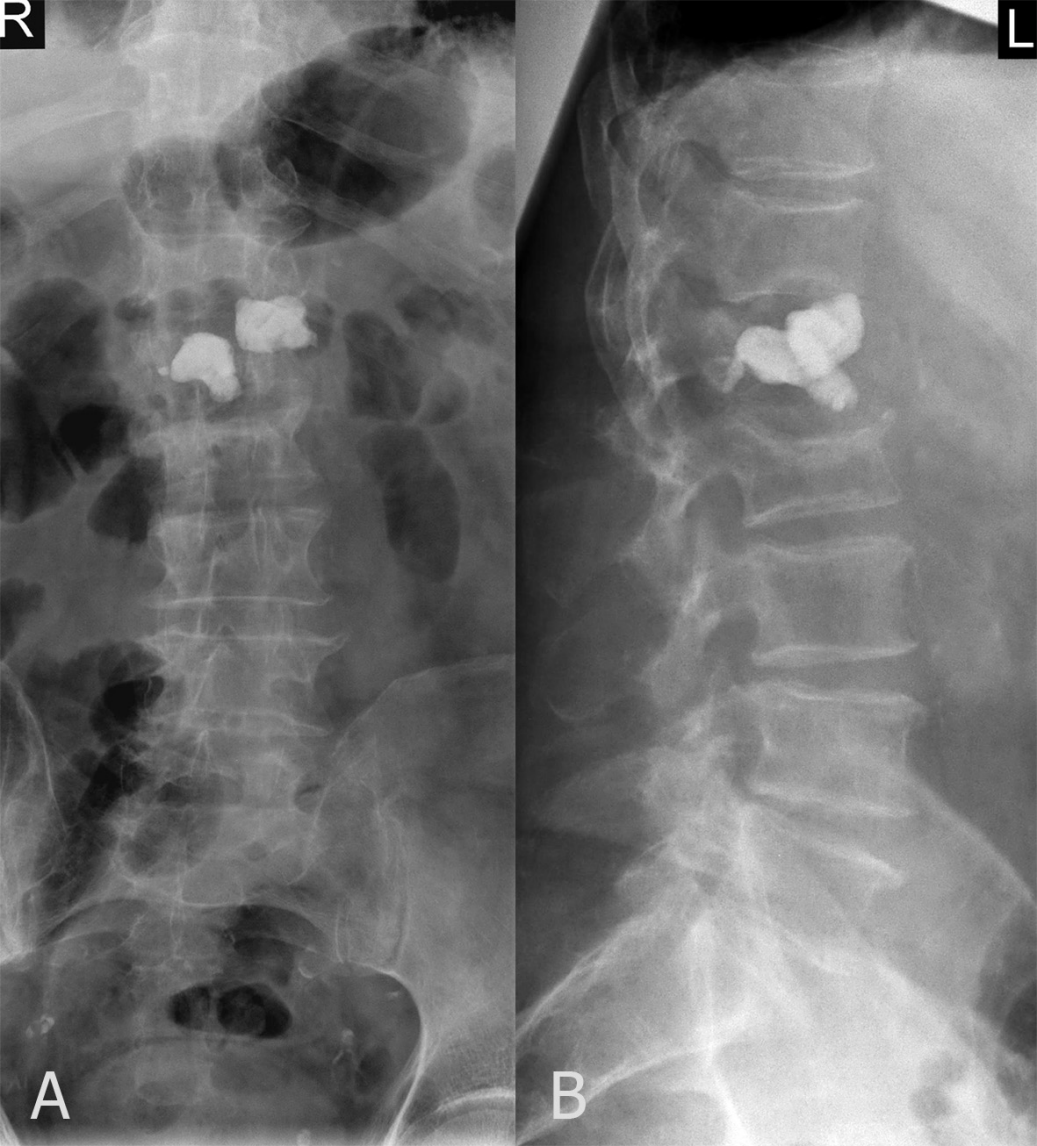
**Anterior posterior (A) and lateral (B) thoracolumbar radiographs (T11 - S1) six weeks after initial kyphoplasty**. The L1 vertebral body is partially resorbed. The osseous structure of the L1 vertebral body cannot be delineated. The position of the left cement block has shifted anteriorly and rostrally.

**Figure 3:**
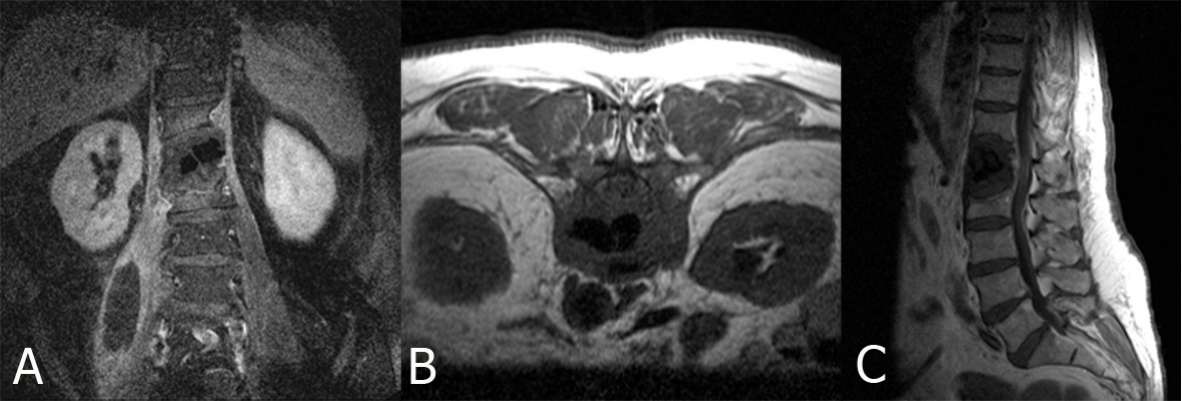
**The magnetic resonance imaging T1 gadolinium-enhanced coronal image (A) shows spondylitis and a right-sided psoas abscess**. T1 without contrast transverse image of L1 (B) demonstrates the compressed spinal canal and inflamed right psoas muscle. T1 sagittal image (C) shows spinal cord compression.

Six weeks after the initial operation, the patient complained of worsening thoracolumbar back pain (Visual Analogue Scale (VAS) 8) requiring hospitalization. On physical examination, incomplete sensory paraplegia below the L1 dermatome was present without motor impairment. The white blood cell count was 14,800 G/L (normal range 4000-10,000 G/L) and the C-reactive protein level was 75 mg/L (normal range 0-5 mg/L). Plain radiographs demonstrated destruction and subtotal resorption of the L1 vertebra, with the cement filling displaced and exposed (Figure 2). In addition, MRI revealed L1 spondylitis with a right-sided psoas abscess and compression of the lumbar spinal cord (Figure 3). These findings were consistent with a diagnosis of pyogenic spondylitis of the L1 vertebra after kyphoplasty.

Re-exploration was recommended but was refused by the patient due to his poor general medical condition, although he was informed about the risk of a progression to complete paralysis. The patient underwent CT-guided aspiration and drainage of the psoas abscess. Cultures grew group C hemolytic *Streptococcus*. He was initially treated conservatively with a six-week course of cefuroxime and clindamycin. The abscess cavity was irrigated daily with normal saline until drain removal on post procedure day six.

**Figure 4:**
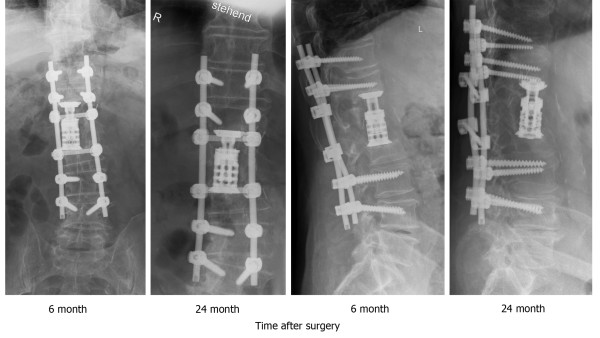
**Anterior posterior (AP) and lateral plain thoracolumbar radiographs six and 24 months after reconstruction and spondylodesis (T11 - L4)**. We performed the transpedicular fixation with a dual rod system and vertebral replacement of the L1 vertebra using an expandable cage. Reconstruction is stable on both AP and lateral views at six months. Follow-up radiographs at 24 months show no signs of pseudarthrosis or infection.

The patient's symptoms progressed to leg paresis without neurogenic bladder and/or bowel dysfunction. He gave informed consent and underwent re-exploration with dorsal spinal decompression, T12/L1 laminectomy and T11 - L4 fusion using transpedicular fixation with a dural rod system (Xia^®^, Stryker Howmedica^®^, Keil, Germany). In a second procedure on postoperative day 10, ventral transphrenic bisegmental spondylodesis was performed. After the removal of the residual L1 vertebra with the cement body, adjacent discs and osteolytic endplates, an intracorporal stand-alone titanium cage (Obelisc, Ulrich Medical, Ulm, Germany) was implanted between T12 and L2. The patient was transferred to the inpatient rehabilitation unit after 11 days. He made an uneventful recovery and his back pain improved significantly (VAS 3). His neurological symptoms regressed after six weeks, with normal biochemistry and no signs of ongoing inflammation. At discharge, his pain was a VAS 2; six months later, he was symptom free and completely ambulatory without assistance (Figure 4). After 24 months, he had no complaints, neurologic deficit or signs of infection. Plain radiographs demonstrated no pseudarthrosis or dislocation of screws, rods or the cage (Figure 4).

## Discussion

This is the first reported case of an infectious complication after kyphoplasty. Since 1998, kyphoplasty has been gaining popularity for the treatment of symptomatic compression fractures as outcomes have been shown to be good [2,4]. Apart from asymptomatic cement leakage, the morbidity is low. Complications after vertebroplasty are also minimal, although there are 10 published cases of primary pyogenic spondylitis after vertebroplasty (Table 1) [7-15]. Only one of these cases was without a significant past medical history. Three were on immunosuppressive medications, three had diabetes mellitus, three were diagnosed with acute urinary tract infections prior to vertebroplasty and one patient had Child's A cirrhosis of the liver secondary to prolonged alcohol abuse [8,11-13]. In addition, one patient had a grade II decubitus ulcer [12]. In four, treatment was conservative without surgical intervention [9-12]. The remaining six patients underwent re-exploration to remove residual material and achieve further stabilization [7,12-15]. One patient with pyogenic spondylitis of T12 following T11 vertebroplasty was treated with drainage at T12 and subsequent vertebroplasty using antibiotic cement [8].

**Table 1:** Literature review of 10 reported cases of pyogenic spondylitis following vertebroplasty.

Author	Affected vertebral body	Side diagnosis	Age	Bacterium	Therapy	Time from vertebroplasty until infection
Deramond [9]	Unstated	Immunosuppressive therapy	Unstated	No detection	Conservative	Unstated

Kallmes [10]	T12	Immunosuppressive therapy	Unstated	Staphylococcus epidermidis	Conservative	1 month

Yu [14]	T12	Urinary tract infection	78	No detection	Dorsoventral stabilization	1 month

Walker [13]	T11 and T12	Urinary tract infection, cholecystitis, meningitis, diabetes mellitus	64	Enterobacter species	Dorsoventral stabilization	11 days

Walker [13]	L3	Discectomy after spondylodiscitis T12/L1	49	*Staphylococcus aureus*	Dorsoventral stabilization	8 months

Schmid [11]	L3 - L5	Liver cirrhosis, alcohol abuse	55	No detection	Conservative	2 weeks

Alfonso [7]	L3	None	63	*Serratia marcescens, Stenotrophmonas maltophilia, Burkholderia cepacia*	Dorsoventral stabilization	1 month

Vats [12]	L1	Diabetes mellitus, decubital ulcus II	73	*Streptococcus agalactiae*	Conservative	6 months

Lin [15]	T12	Immunosuppressive therapy, urinary tract infection	65	Acinetobacter species	Ventral stabilization	6 months

Chen [8]	T11	Diabetes mellitus, vertebroplasty T12	95	*Proprioni acnes*	Drainage with subsequent vertebroplasty	2 months

There is no established evidence as to why more infectious complications have been observed in vertebroplasty versus kyphoplasty. However, the incidence of infectious complications may be attributable to comorbidities, suggesting that high-risk patients may need specific prophylactic antibiotic treatment in order to avoid pyogenic spondylitis. Before our patient's initial kyphoplasty, preoperative imaging and blood tests did not indicate an infectious source in the vertebral body; the bone cylinder biopsy did not show signs of malignancy or infection. Therefore, it is unlikely that an infection that caused the spondylitis was already present. Although the patient had a history of Parkinson's disease and coronary artery disease, these are not regarded as contraindications to kyphoplasty. However, postoperative morbidity may be increased with these comorbidities. One possible cause for an iatrogenic pyogenic infection could be contamination from skin flora [16]. Pyogenic spondylitis and spondylodiscitis following spinal anesthesia have been reported and this may have been the case in our patient; if so, a single dose antibiotic prophylaxis with a first-generation cephalosporin may have been inadequate. To date, there are no official guidelines for antibiotic prophylaxis in spinal surgery.

The cement traditionally used in kyphoplasty does not contain antibiotics. However, the increasing use of antibiotic cement in endoprosthetic surgery is documented. The use of antibiotic cement must be evaluated bearing in mind a patient's individual risk factors, such as age and comorbidities. In immunocompromised patients, the use of antibiotic cement and prolonged perioperative antibiotic prophylaxis should be considered in order to avoid infectious complications. In our case, we propose that there may be a benefit from the use of antibiotic cement in spine augmentation. This area requires further investigation with controlled studies.

In addition, early and emergent spinal cord decompression of the spinal cord is the standard of care. Conservative treatment in this situation is not ideal but we were limited by the patient's refusal to proceed with our initial recommendations. In this case, the primary presenting symptom was recurrent severe back pain. Therefore, severe back pain after a pain-free interval following kyphoplasty must be investigated in order to rule out pyogenic spondylitis. Another diagnosis in the differential that should be considered in such a scenario, especially without adjacent segment fractures, is vertebral necrosis associated with cement injection.

## Conclusion

Complications following kyphoplasty are rare, especially compared with the number of surgeries performed. In pyogenic spondylitis, treatment is laborious and extends over a long period, often involving multiple surgeries. In elderly patients and those with multiple comorbidities, pyogenic spondylitis can be life-threatening. Therefore, antibiotic prophylaxis is likely to be extremely important for the prevention of infectious complications following kyphoplasty in high-risk patients. In these patients, antibiotic cement should be considered.

## Abbreviations

T11: eleventh thoracic vertebra; T12: twelfth thoracic vertebra; L1: first lumbar vertebra; L2: second lumbar vertebra; L3: third lumbar vertebra; L4: fourth lumbar vertebra; CT: computed tomography; MRI: magnetic resonance imaging; VAS: visual analog scale.

## Competing interests

The authors declare that they have no competing interests.

## Consent

Written informed consent was obtained from the patient for publication of this case report and any accompanying images. A copy of the written consent is available for review by the Editor-in-Chief of this journal.

## Authors' contributions

MDS, SL, CDP, TJH and MQ analyzed and interpreted the patient data. MDS performed the surgery. MDS and MQ were the main authors of the manuscript. All authors read and approved the final manuscript.
